# Genomic Characteristics of the Toxic Bloom-Forming Cyanobacterium *Microcystis aeruginosa* NIES-102

**DOI:** 10.7150/jgen.40978

**Published:** 2020-01-01

**Authors:** Haruyo Yamaguchi, Shigekatsu Suzuki, Yasunori Osana, Masanobu Kawachi

**Affiliations:** 1 Center for Environmental Biology and Ecosystem Studies, National Institute for Environmental Studies, 16-2 Onogawa, Tsukuba, Ibaraki 305-8506, Japan.; 2 Department of Electrical and Electronics Engineering, University of the Ryukyus, 1 Senbaru, Nishihara-cho, Okinawa 903-0213, Japan.

**Keywords:** algal bloom, cyanobacteria, genome, microcystin, *Microcystis aeruginosa*, Lake Kasumigaura

## Abstract

*Microcystis aeruginosa*, a bloom-forming cyanobacterium distributed mainly in freshwater environments, can be divided into at least 12 groups (A-K and X) based on multi-locus phylogenetic analyses. In this study, we characterized the genome of microcystin-producing *M. aeruginosa* NIES-102, assigned to group A, isolated from Lake Kasumigaura, Japan. The complete genome sequence of *M. aeruginosa* NIES-102 comprised a 5.87-Mbp circular chromosome containing 5,330 coding sequences. The genome was the largest among all sequenced genomes for the species. In a comparison with the genome of *M. aeruginosa* NIES-843, which belongs to the same group, the microcystin biosynthetic gene cluster and CRISPR-Cas locus were highly similar. A synteny analysis revealed small-scale rearrangements between the two genomes. Genes encoding transposases were more abundant in these two genomes than in other *Microcystis* genomes. Our results improve our understanding of structural genomic changes and adaptation to a changing environment in the species.

## Introduction

Toxic cyanobacterial blooms commonly occur in freshwater environments worldwide. During the summer, these blooms result in serious environmental problems, such as bad odors, bottom-layer anoxia, and cyanotoxin production. *Microcystis aeruginosa* is a unicellular, colony-forming cyanobacterium distributed primarily in eutrophic freshwater environments 1. It is the most well-known toxic bloom-forming cyanobacteria; some strains produce hepatotoxic cyanotoxins called microcystins, which are the only cyanotoxins for which the World Health Organization has established guideline values for drinking water 2. Global climate change, including global warming, is expected to increase the frequency of *Microcystis* blooms 1. *Microcystis* has been a focus of research related to global climate change and the eutrophication of freshwater lakes.

Tanabe et al. classified *M. aeruginosa* isolates by a multi-locus phylogenetic analysis based on seven housekeeping genes and showed that the species has high intraspecific genetic diversity 3. Using this approach, *M. aeruginosa* isolates can be divided into at least 12 phylogenetic groups (A-K and X). The strains in groups A and X and some strains in group B produce microcystins 3, 4.

To date, 4 complete, 22 scaffold-level, and 31 contig-level genome sequences of *M. aeruginosa* have been registered in the National Center for Biotechnology Information Genome database (https://www.ncbi.nlm.nih.gov/genome/genomes/820). *M. aeruginosa* NIES-87, 98, 298, 843, 2481, and 2549 were isolated from a shallow, hyper-eutrophic lake, Lake Kasumigaura, in Japan 5-10, where algal blooms occur every summer to fall 11. *M. aeruginosa* in Lake Kasumigaura has high genetic diversity 12, emphasizing the important of additional sequence information for strains in the lake.

*Microcystis aeruginosa* NIES-102 was collected from Lake Kasumigaura in 1982. A previous phylogenetic analysis has shown that this strain belongs to group A 12. *M. aeruginosa* NIES-102 is of particular interest owing to its production of microcystins, mainly microcystin RR 13. In addition, microviridin, a protease inhibitor produced by several cyanobacteria, was first discovered in this strain 14. In this study, we report the complete genome sequence of *M. aeruginosa* NIES-102 and the results of a comparative genomic analysis with other *M. aeruginosa* genomes.

## Materials and Methods

An axenic culture of *M. aeruginosa* NIES-102 was obtained from the Microbial Culture Collection at the National Institute for Environmental Studies, Japan (http://mcc.nies.go.jp/). DNA extraction from a 20 mL culture of *M. aeruginosa* NIES-102 was performed using NucleoBond Buffer Set III and NucleoBond AXG 100 (Macherey-Nagel, Düren, Germany), following the manufacturer's instructions. DNA sequencing was performed using a MinION sequencer (Oxford Nanopore Technologies, Oxford, UK) and Illumina MiSeq (San Diego, CA, USA). For MinION sequencing, a DNA library was prepared using the Rapid Sequencing Kit (SQK-RAD001) following standard protocols provided by Oxford Nanopore Technologies. The MinION MK1 sequencer and flow cell (R9.4.1) were used for sequencing. In total, 118,979 reads (656,208,396 bp) were obtained. For Illumina MiSeq sequencing, DNA was fragmented using the Covaris M220 Ultrasonicator (Woburn, MA, USA) to obtain 550-bp reads. The DNA library was prepared using the NEBNext Ultra DNA Library Prep Kit for Illumina (New England Biolabs, Ipswich, MA, USA) following the manufacturer's protocol. Sequencing was performed using the 600-cycle MiSeq Reagent Kit v.3. In total, 1,742,106 paired-end reads (949,209,678 bp in total) were obtained. Error correction for nanopore reads was performed using Nanocorr 0.01 15. The corrected nanopore reads were assembled into a single contig using Canu v.1.5 16. The corrected reads were aligned to the contig using BWA-MEM 0.7.17 with a default option 17. The contig was polished using Pilon 1.22 18. The genome was annotated using DFAST 19 with CyanoBase 20 as organism-specific database. A chromosome map of this strain was drawn using DNAPlotter 21. Secondary metabolites were predicted using antiSMASH 22 with default settings. Clustered regularly interspaced short palindromic repeat (CRISPR) loci were detected using CRISPRCasFinder 23. Furthermore, *cas* genes were identified using eggNOG-mapper v.2 24 and BLASTP 25. Functional annotation was performed using eggNOG-mapper v.2 24. Synteny was analyzed using Murasaki 26. The localization of transposases was evaluated using CGView 27.

## Results and Discussion

**Figure 1:**
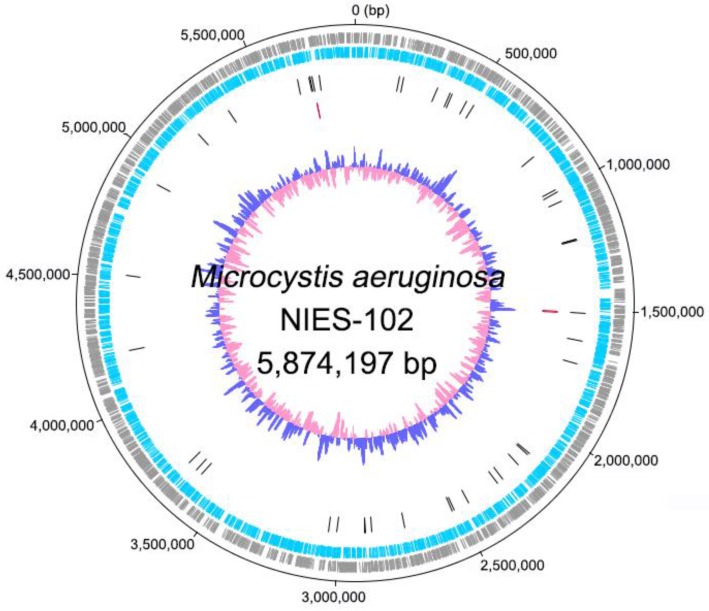
** Complete chromosome map of *Microcystis aeruginosa* NIES-102.** The chromosome map comprises five concentric circles. The gray and light-blue circles show the positions of protein-coding genes on the plus and minus strands, respectively. Black bars on the third circle, red bars on the fourth circle, and blue/pink circle show tRNA, rRNA genes, and guanine-cytosine content.

Genomic characteristics of *Microcystis aeruginosa* NIES-102 are summarized in Table 1. We obtained a genome consisting of a 5.87-Mbp circular chromosome (Fig. 1). Nanopore MinION and Illumina MiSeq read coverages were 112-fold and 162-fold, respectively. The genome of *M. aeruginosa* NIES-102 was the largest among complete genomes of *M. aeruginosa*. It included 5,330 protein-coding sequences, 44 tRNA genes, and two sets of rRNA genes. The G+C content was 42.39%. As the result of GC skew analysis, origin of the replication could not be identified. Using antiSMASH 5.0.0 for prediction, we identified 11 secondary metabolite gene clusters, including microcystin 28, microviridin B 29, aeruginosin 30, and micropeptin biosynthetic gene clusters 31. CRISPRCasFinder predicted a single CRISPR-Cas locus with strong support in the genome with a length of 3,437 bp. The consensus CRISPR repeat sequence was 5′-GTTCCAATTAATCTTAAACCCTATTAGGGATTGAAAC-3′ (37 bp) and there were 47 spacers. According to an established classification system for CRISPER-Cas 32, the locus was subtype I-D CRISPR-Cas 2, consisting of eight genes (*cas3*, *csc3*/*cas10d*, *csc2*, *csc1*, *cas6*, *cas4*, *cas1*, and *cas2*).

**Figure 2:**
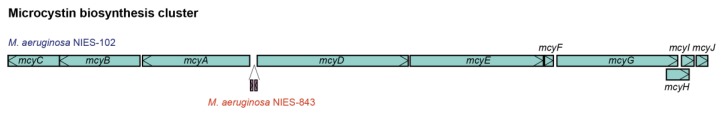
** Comparison of microcystin biosynthesis clusters between *M. aeruginosa* NIES-102 and NIES-843.** The microcystin biosynthesis cluster of *M. aeruginosa* NIES-843 differs from that of NIES-102 in having two additional genes between *mcyA* and *mcyD.*

**Figure 3:**
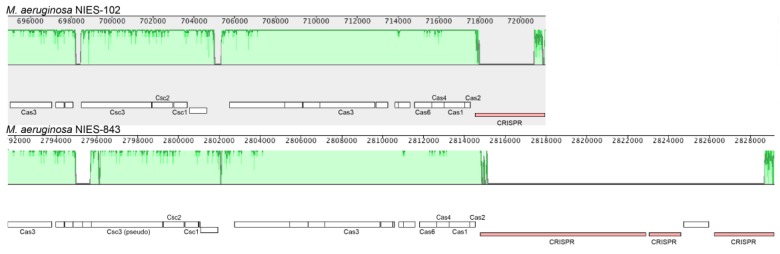
** Comparison of the CRISPR-Cas locus between* M. aeruginosa* NIES-102 and NIES-843.** The genomes of *M. aeruginosa* NIES-102 and NIES-843 have a subtype I-D CRISPR-Cas locus, although the numbers and positions of inserted genes in CRISPR associated genes differ between the two genomes. Only CRISPR-Cas related genes and CRISPR are indicated. The figure was drawn using Mauve software (http://darlinglab.org/mauve/mauve.html).

We compared the genome of *M. aeruginosa* NIES-102 with those of other *M. aeruginosa* strains. The genomes of *M. aeruginosa* NIES-102 and *M. aeruginosa* NIES-843 (group A) shared similar sizes as well as numbers and kinds of genes (Table 1 and 2). The genomes both possess two rRNA operons and the 16S rRNA gene sequences shared 99.7% similarity (5/1485 bp differences). The two strains had similar microcystin biosynthetic gene clusters (Fig. 2); however, two hypothetical proteins were inserted between *mcyA* and *mcyD* in *M. aeruginosa* NIES-843. The similarity of *mcy* genes between *M. aeruginosa* NIES-102 and NIES-843 were 99% excluding *mcyF*, *mcy*H, *mcyJ* (100%) and *mcyD* (98%). Four types of CRISPR-Cas systems have been reported in *M. aeruginosa* 32. The CRISPR-Cas locus in each strain was classified as subtype I-D. However, the numbers and positions of genes in the CRISPR-associated gene clusters differed between the two strains (Fig. 3). These results suggested that the *M. aeruginosa* NIES-102 genome has similar characteristics to those of the *M. aeruginosa* NIES-843 genome, reflecting their close phylogenetic relationship 3.

**Figure 4:**
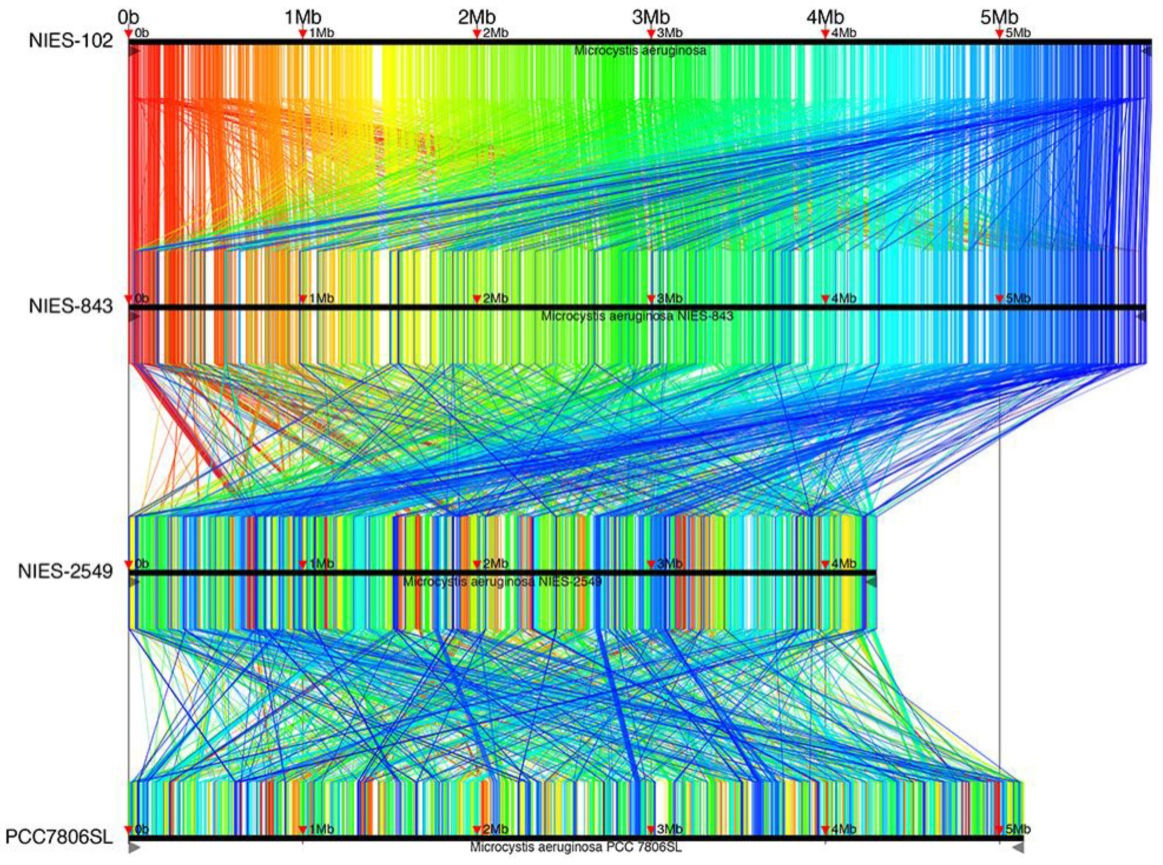
** Synteny analysis of *M. aeruginosa* NIES-102, NIES-843, NIES-2549 and PCC 7806SL.** Similar genomic regions in the four genomes are indicated with the same colors and lines.

Complete genomes of *M. aeruginosa* have been reported for strains NIES-2481, NIES-2549, and PCC7806SL 33 in addition to NIES-843; *M. aeruginosa* NIES-2481 and NIES-2549 are assigned to group G, but *M. aeruginosa* PCC7806SL is unclassified. To identify genomic rearrangements, we conducted a synteny analysis using these strains (Fig. 4). 9,806 conserved regions of length 34-6,489 bp are shown in Fig. 4. The results are filtered by tf-idf scoring feature of Murasaki to remove sequences of high occurrence frequency such as repeat sequences: every region is expected to be highly specific even if the length is as short as 34 bp. The general genomic structures of *M. aeruginosa* NIES-102 and NIES-843 were conserved, with small rearrangements scattered throughout. This result also supports the close relationship between these two strains. We detected frequent recombination between *M. aeruginosa* NIES-843 and NIES-2549 and between *M. aeruginosa* NIES-2549 and PCC7806SL, suggesting substantial divergence between these strains. These results revealed high genomic plasticity in *M. aeruginosa*.

**Figure 5:**
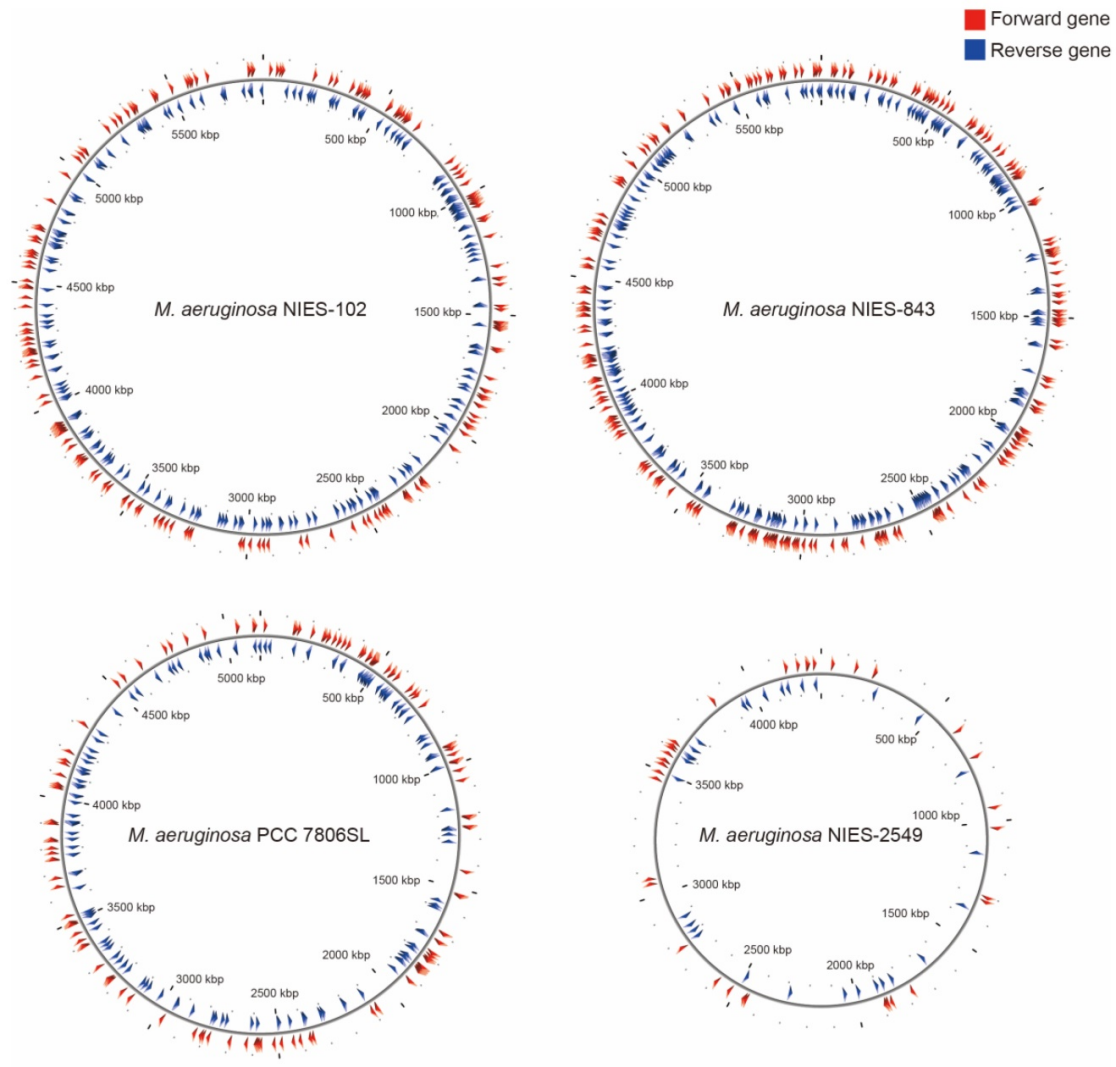
** Localizations of transposases in *M. aeruginosa* NIES-102, NIES-843, NIES-2549 and PCC 7806SL.** Red arrowheads indicate forward genes, and blue arrowheads indicate reverse genes.

Among *M. aeruginosa* strains with complete genomes, *M. aeruginosa* NIES-102 (5.8 Mb) had the largest genome and *M. aeruginosa* NIES-2549 (4.3 Mb) had the smallest genome. The species clearly exhibits diversity in genome size. Yamaguchi et al. 9 suggested that the genome size difference between group A (NIES-843) and group G (NIES-2549) can be partly explained by a difference in the number of genes involved in replication, recombination, and repair (category L, COG). We performed functional annotation using eggNOG-mapper v. 2 against *M. aeruginosa* NIES-102 and NIES-843 genomes (Table 2). The number of orthologous groups assigned to category L in *M. aeruginosa* NIES-102 was similar to that in *M. aeruginosa* NIES-843, suggesting that strains in group A share a large number of genes in category L. Within category L, transposases contribute substantially to variation in genome size. Humbert et al. (2013) showed that the *M. aeruginosa* genome includes a high proportion of genes encoding transposases, providing a basis for rapid divergence and survival in harsh freshwater environments 34. We found that the transposase-coding genes in* M. aeruginosa* NIES-102 and NIES-843 were scattered at a high density throughout the genomes (Fig. 5). In *M. aeruginosa* PCC7806SL, the density of transposases was lower than those in *M. aeruginosa* NIES-102 and NIES-843. *M. aeruginosa* NIES-2549 had the fewest transposases among the four genomes. We detected far more genes encoding transposases in group A than in group G, and these genes may contribute to expansions and contractions of *M. aeruginosa* genomes. Additional genomic analyses are needed to explain the high number of transposes in group A.

In Japanese lakes, including Lake Kasumigaura, *M. aeruginosa* group A is frequently observed 12. The high frequency of strains in group A may be explained by the abundance of genes related to environmental adaptation, such as transposases, in this group. Since freshwater environments change drastically, these genes may promote survival. Climate change and global warming are expected to result in frequent occurrences of algal blooms. Additional genomic information for *M. aeruginosa* would improve our understanding and management of freshwater ecosystems in Japan.

## Figures and Tables

**Table 1:** General characteristics of *M. aeruginosa* NIES-102 and NIES-843

Features	NIES-102 (this study)	NIES-843 (Kaneko et al. 2008)
Genome size (bp)	5,874,197	5,842,795
G+C content (%)	42.39	42.33
Coding sequence (CDS)	5,330	5,897
rRNA operon	2	2
tRNA genes	44	42
Locality	Lake Kasumigaura, Japan	Lake Kasumigaura, Japan
Date of collection	Sep. 1982	Aug. 1997

**Table 2:** Clusters of orthologous group categories of *M. aeruginosa* NIES-102 and NIES-843

Category	Definition	NIES-102	NIES-843
**Cellular processes and signaling**			
D	Cell cycle control, cell division, chromosome partitioning	84	88
M	Cell wall/membrane/envelope biogenesis	228	226
N	Cell motility	70	79
O	Post-translational modification, protein turnover, and chaperones	184	181
T	Signal transduction mechanisms	178	177
U	Intracellular trafficking, secretion, and vesicular transport	82	85
V	Defense mechanisms	72	72
W	Extracellular structures	1	1
Z	Cytoskeleton	1	0
**Information storage and processing**			
A	RNA processing and modification	3	6
B	Chromatin structure and dynamics	1	1
J	Translation, ribosomal structure and biogenesis	197	198
K	Transcription	195	199
L	Replication, recombination and repair	828	880
**Metabolism**			
C	Energy production and conversion	227	228
E	Amino acid transport and metabolism	210	207
F	Nucleotide transport and metabolism	99	99
G	Carbohydrate transport and metabolism	127	132
H	Coenzyme transport and metabolism	175	173
I	Lipid transport and metabolism	82	91
P	Inorganic ion transport and metabolism	183	185
Q	Secondary metabolites biosynthesis, transport, and catabolism	110	120
**Poorly characterized**			
S	Function unknown	1314	1272
